# Hip Arthroplasty Instability After Implantation of a Spinal Cord Stimulator

**DOI:** 10.5435/JAAOSGlobal-D-20-00004

**Published:** 2020-07-02

**Authors:** Cambize Shahrdar, Kevin P. Smidt

**Affiliations:** From the Willis-Knighton Health System Department of Orthopedic Surgery (Dr. Shahrdar), and the Louisiana State University Health Sciences Center Department of Orthopaedic Surgery (Dr. Smidt), Shreveport, LA.

## Abstract

A 46-year-old man with a left hip resurfacing that had been stable for over 5 years sustained a hip dislocation immediately after the implantation of a spinal cord stimulator (SCS). He continued to experience multiple episodes of instability after this initial event, requiring several revision hip arthroplasty surgeries with variable degrees of constraint. It was not until after SCS removal and prolonged hip spica casting that the patient returned to pain-free, independent ambulation. SCS implantation may affect spino-pelvic stability and alter the biomechanics of the hip after hip arthroplasty procedures. We present the unique case of a patient with a well-fixed hip resurfacing with no previous episodes of instability who experienced dislocation immediately after SCS implantation.

Traditional hip replacement can be inherently unstable. An anatomic skeletal femoral head varies in diameter, and on average, a female femoral head is 42.2 mm and a male femoral head is 48.4 mm.^1^ Hip replacements originally used femoral head diameters that were in the range of 22 mm in the 1960s, increasing to 28 mm in the 1990s and up to 32 mm in the mid-2000s.^2^ After the advent of highly cross-linked polyethylene, prosthetic femoral head diameters increased to 36, 38, 40, and 44 mm. Studies on these larger femoral head sizes have demonstrated a decreased incidence of dislocation.^3-5^ One of the effects of using a larger femoral head diameter is that it increases the jump distance or degree of translation of the femoral head center before dislocation occurs.^6,7^ With hip resurfacing, hip surgeons are able to resurface a femoral head to on average −2 mm of the native femoral head minimum diameter measurement.^8,9^ For example, if during a hip resurfacing, the native femoral head minimum diameter is 46 mm, then the final femoral head implant size would be 44 mm in diameter. Because a hip resurfacing procedure most closely maintains the biomechanical features of the native hip, it is generally felt that it is a more anatomic form of hip reconstruction and inherently as stable as the original hip.^9-11^

In 2006, there was a renewed interest in metal-on-metal (MoM) bearing surfaces in hip arthroplasty due to the decreased risk of dislocation with larger head sizes and the idea that MoM would have less wear than metal-on-polyethylene bearing surfaces.^10,11^ Further studies exposed the risks and complications associated with MoM hip replacements, and this had led to a dramatic decline in the number of MoM hips implanted in the United States over the past few years.^9,12^

The question of hip stability continues to be studied, and we are still learning more and more regarding this phenomenon.^2,4,7,9,13,14^ Recently, publications are alluding to a correlation between the spine and hip stability, particularly the angle of the pelvis relative to the spine in patients with a history of lumbar spinal fusion surgery.^15,16,17,18,19^ Resultant spino-pelvic stiffness after lumbar fusion interferes with the ability of the pelvis to flex in a seated position, causing the hip to remain in a less anteverted position. In a patient with a hip replacement, this can result in posterior dislocation of the prosthesis.

It is not uncommon for the aging population to develop both hip and spine involvement in the arthritic process.^20,21^ Patients can present with pain in the lower back, buttocks, and groin and can also have leg pain with radiating symptoms. The consensus is to always treat the hip first, unless there is concern of imminent paralysis from severe spinal stenosis.^21-23^ The concept of spino-pelvic balance is equally as important as recognizing that nerve compression and an imbalance of the hip musculature can cause weakness in the posterior hip muscles, resulting in hip dislocation.^9-11^

Spinal cord stimulation was devised as a way to help alleviate symptoms in patients who suffer from chronic pain in the spine or extremities and lessen the use of narcotic pain medications and their associated complications.^24-26^ A spinal cord stimulator (SCS) is a surgically implanted device that places an electrode near the spinal cord which is connected to a control unit or generator. The stimulating electrode will then prevent pain signals from being transmitted to the brain. This system has been extensively studied, and published data have suggested a success rate of 50% to 70% in relieving symptoms in patients with chronic pain.^25,26,27,28^ Nearly 60,000 SCS units are implanted each year in the United States.^29-31^ However, there have been numerous accounts of injury as a result of SCS units, and they currently rank third among all injury reports to the FDA for surgically implanted devices.^31^ Known risks and complications of SCS include device malfunction, shocks, burns, infection, spinal cord nerve damage, muscle weakness, and paraplegia.^32,33^

Currently, there is no known publication that has recognized the association of SCS with the risk of hip arthroplasty dislocation. We present a case to inform the orthopaedic surgical community of this relationship. The patient was informed that the data concerning the case would be submitted for publication, and he provided consent.

## Case Report

**Figure 1:**
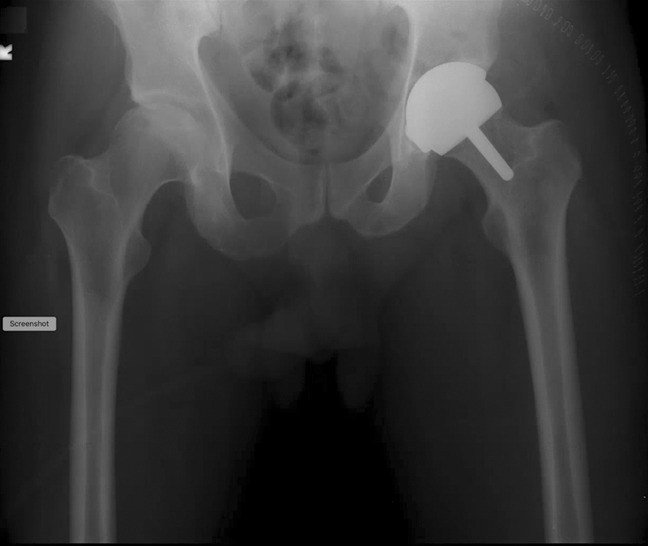
Postoperative radiograph after left Birmingham hip resurfacing procedure.

A 46-year-old man presented with severe bilateral hip and low back pain. The patient was diagnosed with bilateral hip arthritis and osteonecrosis and underwent a successful left Birmingham hip resurfacing through a posterior Kocher-Langenbeck approach (Figure 1). The implant cup abduction angle was 44°. The patient eventually underwent a right total hip replacement and reported no pain in either hip at subsequent follow-up visits.

**Figure 2:**
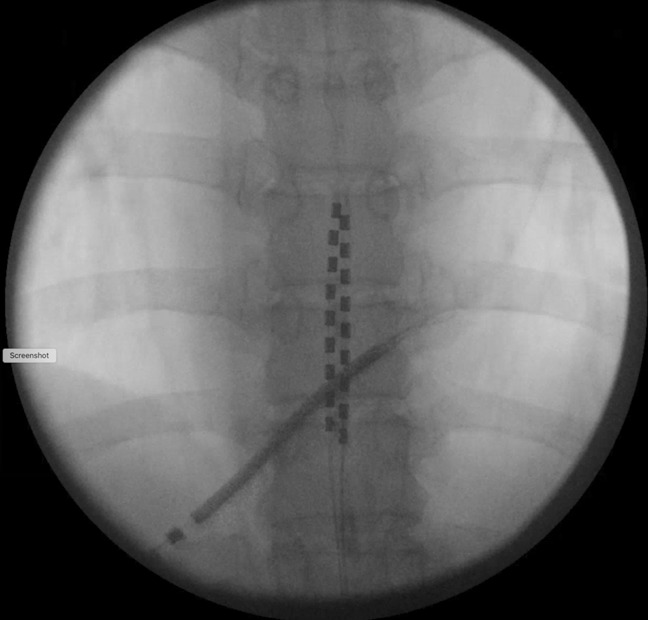
Intraoperative radiograph of the spinal cord stimulation device implantation.

**Figure 3:**
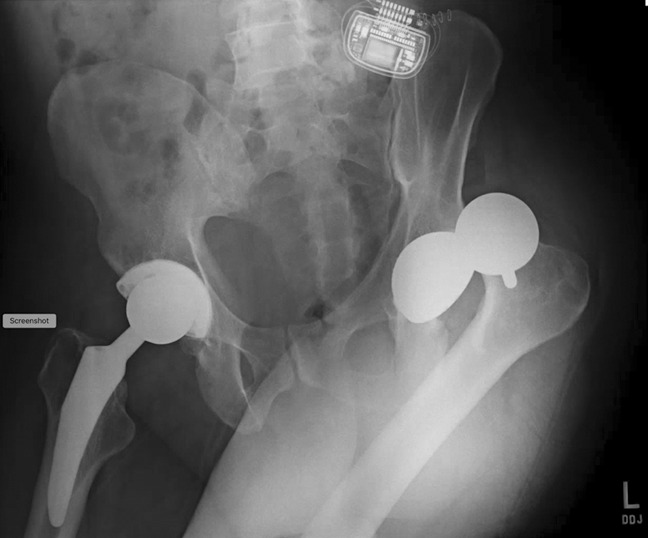
Radiograph revealing dislocation of a well-fixed and functioning Birmingham hip resurfacing immediately after surgical placement of a spinal cord stimulator for pain management of back pain.

Over 5 years after his left hip resurfacing, the patient was still living with chronic back pain and underwent SCS implantation (Figure 2). The SCS was inserted with the patient in the prone position. The patient was then rolled back to the supine position and transported to the postanesthesia care unit. Review of the surgical report revealed no complications during the procedure. In the recovery room, the SCS medical representative arrived and programmed the device with the patient lying in the supine position. Several minutes after the representative finished programming the SCS, the patient pulled himself to sit upright and immediately experienced severe pain in his left hip. A radiograph was taken demonstrating posterior dislocation of his left hip resurfacing (Figure 3).

**Figure 4:**
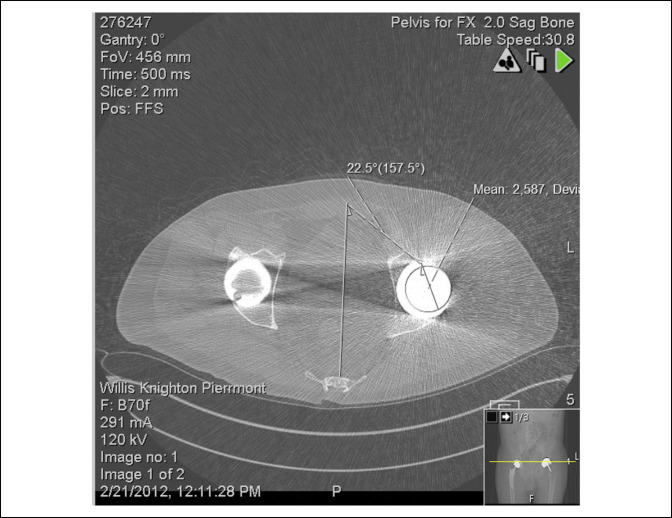
CT scan revealing the left Birmingham hip resurfacing cup anteversion angle of 22.5°.

He was transferred to the nearest emergency department and underwent a successful immediate closed reduction; however, 7 months later, he suffered two more dislocations within a 24-hour period which required subsequent closed reductions. A CT scan was obtained, which revealed the left Birmingham hip cup had 22.5° of anteversion (Figure 4). The patient was counseled regarding surgical versus nonsurgical management of his left hip arthroplasty instability and, given his history of three dislocations and apprehension to ambulate, he elected for revision surgery.

**Figure 5:**
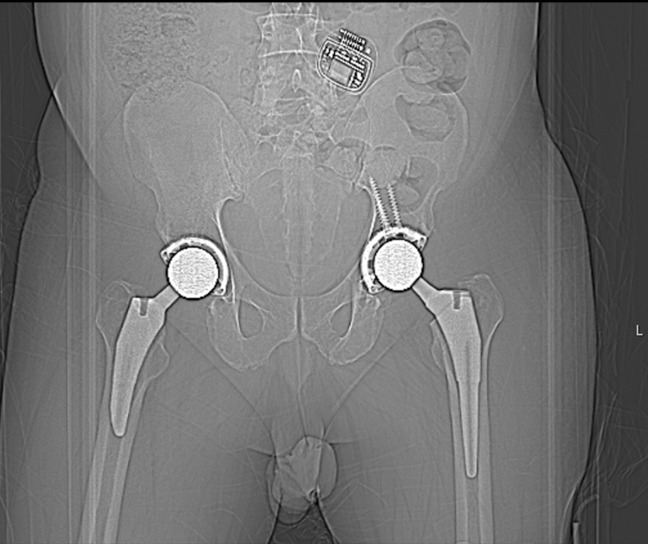
Postoperative radiograph of revision left total hip arthroplasty.

**Figure 6:**
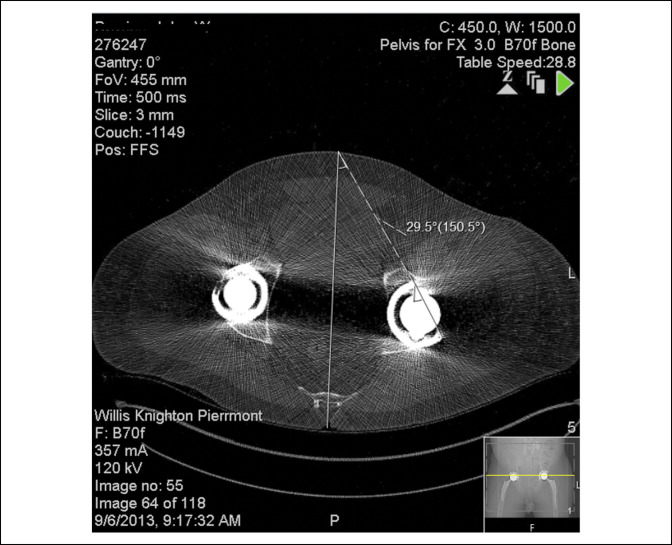
CT scan of the revision left total hip arthroplasty revealing that new cup position is 29.5° of anteversion.

He underwent a left hip revision whereby his hip resurfacing was removed through an anterior approach, and a pressfit shell was implanted with screw fixation and a metal-on-plastic bearing. A pressfit Taperloc stem with a size of 36 mm head was used for the femoral implant (Figure 5). One month after his revision surgery, his left total hip arthroplasty (THA) dislocated posteriorly. Repeat CT scan revealed that his left acetabular anteversion was 29.5° (Figure 6). The hip was reduced but subsequently dislocated again, requiring a reduction under anesthesia. Due to this new recurrent instability, the treating orthopaedic surgeon requested that the SCS be removed by the patient's pain management physician after discussing the complications suffered from the SCS with the patient. The SCS was successfully removed 9 months after it had been implanted.

Two months after the SCS was removed, the patient experienced another left hip posterior dislocation. A decision was made to perform a repeat revision THA because of his persistent instability and apprehension. The 36 mm femoral head was removed, and a constrained liner was implanted. Unfortunately, the patient developed a prosthetic hip infection 1 month later. The left THA was resected, and an articulating antibiotic spacer was placed. Three days later, the cement spacer dislocated posteriorly, and the patient underwent another revision surgery whereby the articulating spacer was replaced with a nonarticulating spacer.

After 6 weeks of intravenous antibiotics followed by a 6 week antibiotic holiday, a new dual-mobility THA was implanted. The patient's perioperative course was uncomplicated until he suffered another posterior hip dislocation 5 weeks later. The hip was successfully reduced but once again dislocated a week later. All of the dislocations suffered by the patient were posterior. Finally, he was re-reduced under anesthesia and placed into a hip spica cast for 3 months. As of his latest follow-up visit nearly 5 years later, he has experienced no further dislocation events. Both hips were pain free at that time, and he was ambulating without the use of any assistive devices.

## Discussion

There have been significant advancements in the understanding of the spino-pelvic relationship because it relates to hip replacement surgery. Hip arthroplasty surgeons must recognize that alterations in spino-pelvic mobility have an impact on the future stability of a hip replacement. It is well recognized in the literature that lumbar fusion is a risk for hip arthroplasty dislocation. The patient presented in this case report had a functioning hip resurfacing that was pain free, adequately positioned, and completely stable for over 5 years. He had experienced no dislocation events until immediately after the implantation of an electrode near his spinal cord, and no complications were reported from the procedure. No reports exist in the medical literature that describe a hip replacement device that has dislocated after the immediate implantation of an SCS used to treat chronic pain. In this patient, even after the SCS was removed, he continued to have hip instability and additional complications due to instability. It was not until 1 year after the SCS was removed and 3 months of immobilization in a spica cast that his hip instability was eradicated. This is the first report of hip arthroplasty instability immediately after implantation of an SCS. It is recommended that orthopaedic surgeons advise patients of this potential complication if they are contemplating SCS implantation in the setting of a well-functioning hip replacement.
